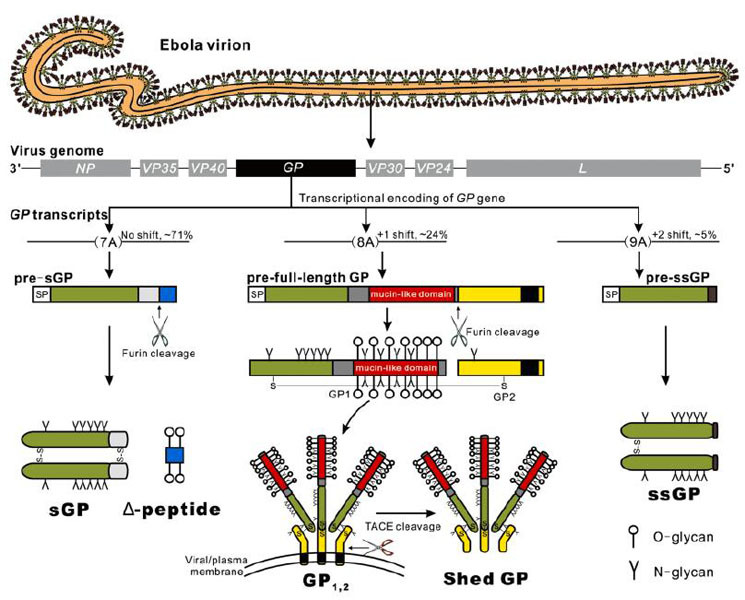# The roles of ebolavirus glycoproteins in viral pathogenesis

**DOI:** 10.1007/s12250-016-3850-1

**Published:** 2016-11-14

**Authors:** Yun-Jia Ning, Fei Deng, Zhihong Hu, Hualin Wang

**Affiliations:** State Key Laboratory of Virology, Wuhan Institute of Virology, Chinese Academy of Sciences, Wuhan, 430071 China

**Keywords:** ebolavirus, glycoprotein (GP), mucin-like domain (MLD), cytotoxicity, immune evasion, inflammation, pathogenesis

## Abstract

Ebolaviruses are highly dangerous pathogens exhibiting extreme virulence in humans and nonhuman primates. The majority of ebolavirus species, most notably *Zaire ebolavirus*, can cause Ebola virus disease (EVD), formerly known as Ebola hemorrhagic fever, in humans. EVD is associated with case-fatality rates as high as 90%, and there is currently no specific treatment or licensed vaccine available against EVD. Understanding the molecular biology and pathogenesis of ebolaviruses is important for the development of antiviral therapeutics. Ebolavirus encodes several forms of glycoproteins (GPs), which have some interesting characteristics, including the transcriptional editing coding strategy and extensive O-glycosylation modification, clustered in the mucin-like domain of GP1, full-length GP (GP_1,2_), and shed GP. In addition to the canonical role of the spike protein, GP_1,2_, in viral entry, ebolavirus GPs appear to have multiple additional functions, likely contributing to the complex pathogenesis of the virus. Here, we review the roles of ebolavirus GPs in viral pathogenesis.

## INTRODUCTION

The 2014 Ebola epidemic in West Africa was caused by the most pathogenic ebolavirus species, EBOV (*Zaire ebolavirus*), and resulted in 28, 652 suspected, probable, and confirmed cases, with 11, 325 deaths as of April 13, 2016 (U.S. Centers for Disease control and Prevention, 2016a). As before the 2014 outbreak, epidemics of highly pathogenic ebolaviruses had primarily occurred sporadically in central Africa, the study of these viruses had been somewhat neglected for many years. The unprecedented and massive damage to human health and the social economy caused by the recent outbreak raised the alarm regarding the potential large-scale re-emergence of such deadly viral diseases. To date, five ebolavirus species, including *Zaire ebolavirus*, *Sudan ebolavirus* (Sudan virus), *Bundibugyo ebolavirus* (Bundibugyo virus), *Taï Forest ebolavirus* (Taï Forest virus), and *Reston ebolavirus* (Reston virus) (Bukreyev et al., 2014) have been identified. In addition to EBOV, Sudan virus, Bundibugyo virus, and Taï Forest virus are also pathogenic to humans, while human infections with Reston virus are thought to be asymptomatic (Peters and Leduc, 1999; Feldmann and Geisbert, 2011; Rougeron et al., 2015; To et al., 2015).

Although many studies of ebolavirus (particularly EBOV) have been performed using animal infection models (Nakayama and Saijo, 2013; Shurtleff and Bavari, 2015), knowledge of the pathology and pathogenesis of human ebolavirus infections remains limited. Highly pathogenic ebolavirus infections in humans and non-human primates are characterized by immune suppression and dysregulated inflammatory responses which can cause uncontrolled virus replication, immune system impairment, and tissue damage and thus likely result in the manifestations of Ebola virus disease (EVD), such as high fever, hemorrhage, disseminated intravascular coagulation, multiorgan failure, and shock (Zaki and Goldsmith, 1999; Mahanty and Bray, 2004; Feldmann and Geisbert, 2011; Kortepeter et al., 2011; Ansari, 2014; Chertow et al., 2014; Singh et al., 2015).

Taxonomically, ebolaviruses are enveloped, negative-sense RNA viruses which belong to the genus *Ebolavirus* of the family *Filoviridae*. Ebolaviruses encode several structural proteins including the nucleoprotein (NP), virion protein 24 (VP24), VP30, VP35, VP40, polymerase (L), and full-length transmembrane glycoprotein ($GP_{1,2}$), as well as multiple nonstructural glycoproteins (GPs) such as the soluble glycoprotein (sGP), Δ-peptide, small soluble glycoprotein (ssGP), and shed GP (Lee and Saphire, 2009; Cook and Lee, 2013) (Figure 1). The production of the various GP forms results from transcriptional editing (mRNA editing by the viral polymerase-mediated addition or deletion of nucleotides at the specific site) of the *GP* gene (Volchkov et al., 1995; Sanchez et al., 1996) and post-translational processing of GP precursors (Figure 1).

$GP_{1,2}$ is the virus surface protein and forms trimeric spikes (Sanchez et al., 1998) that facilitate virus entry by mediating receptor binding and membrane fusion (Lee and Saphire, 2009). In addition to the essential role of $GP_{1,2}$ as the structural protein mediating virus entry, $GP_{1,2}$ as well as other GP variants have also been shown to, or are suspected to, have multiple functions in the virus life cycle and virus-host interactions, thus likely making a versatile contribution to the complex pathogenesis of ebolavirus.

Knowledge of viral protein functions and their contributions to viral pathogenesis will contribute to the development of prophylactic treatment to protect against ebolavirus infections. In this review, the function of ebolavirus GPs is briefly summarized, with a particular focus on their defined, or potential, roles in viral pathogenesis.

## FULL-LENGTH TRANSMEMBRANE GP ($GP_{1,2}$)

Full-length transmembrane GP ($GP_{1,2}$) is the product of the *GP* gene with a + 1 shift of the open reading frame (ORF), resulting from transcriptional editing (Volchkov et al., 1995; Sanchez et al., 1996) (Figure 1). Processing of $GP_{1,2}$ in the endoplasmic reticulum (ER) and Golgi apparatus results in the generation of disulfide-linked GP1 and GP2 subunits, which are furin-cleavage products (Jeffers et al., 2002; Volchkov et al., 1998) of the full-length GP polyprotein precursor (Volchkov et al., 1998a; Volchkov, 1999; Wool-Lewis and Bates, 1999; Ito et al., 2001; Jeffers et al., 2002) (Figure 1). $GP_{1,2}$ heterodimers can form trimeric structures located at the cell surface by the transmembrane domain (TMD) of GP2, or subsequently on the virus surface as virion spikes, which viruses obtain while budding from the plasma membrane (Sanchez et al., 1998; Harty et al., 2000; Han et al., 2003; Panchal et al., 2003) (Figure 1). The surface of mature $GP_{1,2}$ is covered with N- and O-linked glycans (Jeffers et al., 2002; Ritchie et al., 2010; Lennemann et al., 2014); notably, GP1 contains a serine, threonine, and proline-rich mucin-like domain (MLD) of approximately 150 amino acid (aa) residues, which is heavily O-glycosylated at approximately 80 sites (Jeffers et al., 2002).

### The roles of full-length GP ($GP_{1,2}$) in the ebolavirus life cycle

As the virion surface spikes, trimeric $GP_{1,2}$ complexes mediate ebolavirus entry processes, the initial steps of viral infection, with GP1 acting as the receptor-binding subunit and GP2 as the membrane fusion subunit (Jiang et al., 2009; Lee and Saphire, 2009; Wang et al., 2011). The $GP_{1,2}$-mediated entry processes and entry-associated structural and functional characterization of $GP_{1,2}$ have been intensively investigated in ebolavirus and are discussed extensively elsewhere (Lee et al., 2008; Lee and Saphire, 2009; Falzarano and Feldmann, 2015; Moller-Tank and Maury, 2015; Gong et al., 2016; Tang, 2016; Wang et al., 2016; White and Whittaker, 2016). Due to its ability to bind to a variety of cell surface molecules, $GP_{1,2}$ largely determines the broad cellular tropism of ebolaviruses, which can infect a wide range of cell types, including immune cells (monocytes, macrophages, and dendritic cells) and many other tissue cell types (endothelial cells, hepatocytes, adrenal cortical cells, and some epithelial cells, among others) (Feldmann and Geisbert, 2011; Takada, 2012; Martines et al., 2015; Singh et al., 2015). The infection of these cells by ebolaviruses and the resultant disturbance of normal cell physiology undoubtedly have important roles in the complicated pathogenesis of ebolavirus. For example, aside from the disorders of host immune/inflammatory responses caused by the infection of immune cells, infected monocytes, macrophages, and dendritic cells likely contribute to viral spread from the initial site of infection to regional lymph nodes, and the liver, spleen, and other tissues/organs, through the blood and lymphatic systems, especially as these immune cells are early and preferred replication sites of ebolaviruses (Schnittler and Feldmann, 1998; Geisbert et al., 2003; Bray and Geisbert, 2005). Moreover, infections in the liver, and the resulting hepatocellular necrosis, could impair synthesis of coagulation factors and other plasma proteins, contributing to the hemorrhagic tendencies observed in infected individuals, while adrenocortical infection and necrosis could damage adrenocortical function and inhibit steroid synthesis, promoting the development of hypotension, hypovolaemia, and shock that are often noted in EVD cases.

Figure 1. Encoding strategy of ebolavirus GPs. Ebolaviruses are enveloped, negative-sense RNA viruses belonging to the family *Filoviridae*. The ebolavirus genome contains seven genes (3'-*NP*-*VP35*-*VP40*-*GP*-*VP30*-*VP24*-*L*-5'), among which the *GP* gene encodes three GP precursors, resulting in multiple GP protein products. The primary product of the *GP* gene is pre-sGP, which is expressed from the majority of RNA transcripts (e.g., approximately 71% of the total transcripts in EBOV-infected Vero E6 cells) with no shift of ORF and can be cleaved by furin at its C-terminus, yielding an N-glycosylated sGP dimer and an O-glycosylated Δ-peptide. Transcriptional editing can occur at a series of seven uridine residues in the *GP* gene, resulting in corresponding changes in the number of adenosine (A) residues in the transcripts. A + 1 shift results in an extended ORF, encoding the full-length GP (approximately 24% of transcripts), while a + 2 shift leads to the synthesis of the ssGP from the truncated ORF (approximately 5% of transcripts). Note that the addition of more A residues or the deletion of a single A nucleotide has also been observed, although transcripts containing 7A, 8A, or 9A at the transcriptional editing site are the most common, and encode sGP, full-length GP, or ssGP, respectively. The full-length GP precursor is cleaved by furin to form a disulfide-linked GP1-GP2 dimer, which subsequently assembles into the $GP_{1,2}$ trimer and locates to the plasma or viral membrane. $GP_{1,2}$ trimers on the viral membrane function as virion surface spikes, facilitating virus entry. A feature of $GP_{1,2}$ is that it contains not only N-glycans, but also extensive O-glycans, clustered in the mucin-like domain (MLD) of the GP1 subunit. As reflected in the diagram, in the native structure of GP1, the MLD (red) sits above the N-terminal domain. Some surface $GP_{1,2}$ can be further cleaved by the TNF-α converting enzyme (TACE) at the membrane-proximal external region to release shed GP. Like sGP, mature ssGP also forms a dimer and is N-glycosylated. ssGP, full-length GP (specifically GP1), and sGP share a common N-terminus (green), but differ in their C-termini, thus exhibiting diverse functions.

The innate immune system, and particularly the type I interferon (IFN) response, provides a stubborn line of defense in resistance to viral infections. IFN induction and signaling lead to the establishment of the host antiviral state by inducing the expression of more than 300 IFN-stimulated genes which can limit viral replication and spread by targeting multiple aspects of the viral life cycle (Stark, 2007; Randall and Goodbourn, 2008; Sadler and Williams, 2008; Schneider et al., 2014; Errett and Gale, 2015). However, viruses, especially highly pathogenic hemorrhagic fever viruses, have evolved various strategies to antagonize these antiviral responses (Elliott and

Weber, 2009; Borrow et al., 2010; Ye et al., 2013; Ning et al., 2014; Ma and Suthar, 2015; Messaoudi et al., 2015; Ning et al., 2015). VP35 and VP24 are well-known IFN antagonists of ebolaviruses (Audet and Kobinger, 2015; Basler, 2015; Messaoudi et al., 2015), and the full-length GP also exhibits an antagonistic activity against the innate immune system by targeting tetherin, an IFN-induced antiviral factor (Van damme et al., 2008; Kaletsky et al., 2009; Tokarev et al., 2009). Tetherin is a type Ⅱ transmembrane protein containing an N-terminal TMD and a C-terminal glycosylphosphatidylinositol (GPI) membrane anchor, which is considered an unusual topology (Kupzig et al., 2003). Tetherin restricts the release of some enveloped viruses (including ebolaviruses) from infected cells by retaining virions on the cell surface through simultaneous anchoring to both the plasma membrane and the virus envelope (Jouvenet et al., 2009; Perez-Caballero et al., 2009; Tokarev et al., 2009; Fitzpatrick et al., 2010; Hammonds et al., 2010; Hinz et al., 2010; Le tortorec et al., 2011). Ebolavirus full-length GP can counteract the antiviral activity of tetherin, facilitating the completion of virus budding (Kaletsky et al., 2009). Thus, in addition to its essential roles in viral entry, full-length GP also contributes to viral budding, not only by participating in virus assembly as the structural spike glycoprotein, but also by antagonizing tetherin-mediated antiviral activity. Although the mechanism underlying the antagonism of tetherin by full-length GP has not yet been clearly defined, it was reported that the ebolavirus GP does not remarkably effect the expression level of tetherin on the cell surface, or prevent the association of tetherin with lipid rafts, but may block the interaction of tetherin with VP40, a primary matrix protein with a key role in virus assembly and budding, and this may contribute to the observed antagonistic activity (Kaletsky et al., 2009; Lopez et al., 2010; Kuhl et al., 2011; Lopez et al., 2012; Gustin et al., 2015; Vande Burgt et al., 2015).

## The pathogenicity of full-length GP ($GP_{1,2}$) and its MLD

In addition to its pivotal contributions to the virus life cycle, the $GP_{1,2}$ protein itself also exhibits notable direct pathogenicity to host cells. Expression of EBOV $GP_{1,2}$ causes evident rounding and detachment of adherent cells, such as cultured human or non-human endothelial cells, epithelial cells, and macrophages (Chan et al., 2000; Takada et al., 2000; Yang et al., 2000; Simmons et al., 2002). $GP_{1,2}$ from other ebolavirus species can also exert similar effects on cells, albeit at lower levels than EBOV $GP_{1,2}$, paralleling the differences in virulence observed among ebolavirus species (Simmons et al., 2002). Importantly, $GP_{1,2}$ expression from a replication-defective adenoviral vector induces massive endothelial cell detachment in explanted blood vessels and thus substantially increases vascular permeability (Yang et al., 2000). Interestingly, EBOV $GP_{1,2}$ causes endothelial disruption in both human and non-human primate blood vessels, whereas $GP_{1,2}$ from Reston virus damages non-human primate but not human blood vessels, further indicating the role of $GP_{1,2}$ as a virulence factor mediating vascular damage (Yang et al., 2000).

The MLD is required for $GP_{1,2}$-induced toxicity to both cultured adherent cells and blood vessel explants (Yang et al., 2000; Simmons et al., 2002). In addition, cell surface expression of full-length GP, including a TMD, is needed for the $GP_{1,2}$ activity; however, the requirement for a TMD does not appear to be for the specific TMD of GP2, as the toxicity of $GP_{1,2}$ is maintained when the GP2 TMD is substituted with other TMDs (including those derived from influenza virus hemagglutinin, and Moloney murine leukemia virus envelope protein) (Takada et al., 2000; Yang et al., 2000). Moreover, surface expression of the MLD fused with an exogenous TMD is sufficient to induce cytopathic effects (CPE), including cell rounding and detachment (Yang et al., 2000; Francica et al., 2009).

Mechanistic studies have demonstrated that the detectable levels of cellular surface molecules, such as integrins, intercellular cell adhesion molecule-1(ICAM-1), platelet/endothelial cell adhesion molecule-1 (PECAM-1), vascular cell adhesion molecule-1 (VCAM-1), epidermal growth factor receptor (EGFR), and major histocompatibility complex class I (MHC-I) appear to decrease in $GP_{1,2}$-expressing cells (Takada et al., 2000; Simmons et al., 2002). Initially, it was considered that the surface expression of these molecules was down-regulated by $GP_{1,2}$/MLD; however, further studies suggested that MLD, acting as a “glycan umbrella”, sterically shields the epitopes and functions of cellular surface proteins, rather than affecting their abundance (Reynard et al., 2009; Francica et al., 2010). Considering the significant roles of these surface proteins in physiological functions of cells, such as adhesion and immune/inflammatory signaling, $GP_{1,2}$/MLD-mediated cytotoxicity may contribute significantly to the inflammatory dysregulation, immune suppression, and vascular damage characteristics of ebolavirus pathogenesis.

As noted above, it is reasonable to assume that the shielding of surface molecules of endothelial cells explains $GP_{1,2}$-induced endothelial CPE and vascular damage, while the functional impairment of immune cell surface proteins would interfere with the immune and inflammatory responses. Moreover, it has indeed been shown that $GP_{1,2}$ expression blocks CD8+ T cell-recognition of MHC-I on antigen-presenting cells, impairing MHC-I antigen presentation (Francica et al., 2010). Steric shielding of cell surface immune molecules by the massive glycans of MLD is a novel mechanism of viral immune evasion. Furthermore, although antibodies can be raised against the MLD, this region is unnecessary for ebolavirus entry and highly variable compared with the other parts of the full-length GP (Wilson et al., 2000; Jeffers et al., 2002). Interestingly, MLD glycans mask antigenic epitopes of the core structure (including the receptor binding domain) of $GP_{1,2}$ itself, beyond the MLD, and thereby likely blocking the activities of neutralizing antibodies, perhaps representing another MLD-mediated immune escape strategy of ebolaviruses (Lee et al., 2008; Reynard et al., 2009; Francica et al., 2010; Dias et al., 2011; Martinez et al., 2011; Cook and Lee, 2013; Misasi et al., 2016; Pallesen et al., 2016).

In addition to the MLD, a putative immunosuppressive domain (ISD) close to the C-terminus of $GP_{1,2}$ may also contribute to viral immune suppression by inhibiting T cell cycle progression and inducing T cell apoptosis, thus likely promoting the lymphocyte depletion observed in severe infections (Volchkov et al., 1992; Becker, 1995; Baize et al., 1999; Zaki and Goldsmith, 1999; Geisbert et al., 2000; Yaddanapudi et al., 2006).

Clinically, ebolavirus infection is typified by an exaggerated inflammatory response resembling septic shock (Zaki and Goldsmith, 1999; Baize et al., 2002; Cilloniz et al., 2011; Feldmann and Geisbert, 2011; Kortepeter et al., 2011; Ansari, 2014; Singh et al., 2015). Several studies have suggested that $GP_{1,2}$, and in particular the MLD, are likely involved in activation of the inflammatory response (Wahl-Jensen et al., 2005a; Ye et al., 2006; Martinez et al., 2007; Okumura et al., 2010). Virus-like particles (VLPs) harboring $GP_{1,2}$ can activate dendritic cells and macrophages and trigger the secretion of inflammatory cytokines, such as tumor necrosis factor-α (TNF-α), interleukin-6 (IL-6), IL-8, IFN--inducible protein-10 (IP-10), and the chemokine RANTES (regulated upon activation normal T cell expressed and secreted) (Bosio et al., 2004; Wahl-Jensen et al., 2005a; Ye et al., 2006; Martinez et al., 2007; Okumura et al., 2010). Functional studies further demonstrate that $GP_{1,2}$ on VLPs simulates the activation of NF-κB and elicits inflammatory cytokine production via the toll-like receptor-4 (TLR4) signaling pathway. Moreover, MLD is also indispensable for the activity of $GP_{1,2}$ (Martinez et al., 2007; Okumura et al., 2010). In addition to TLR4, the liver and lymph node sinusoidal endothelial cell C-type lectin (LSECtin) may also be involved in $GP_{1,2}$-triggered pro-inflammatory cytokine production in dendritic cells, by recognizing $GP_{1,2}$ as an additional pattern recognition receptor and activating 12 kDa DNAX-activating protein (DAP12)-Syk signaling (Zhao et al., 2016). These effects, mediated by $GP_{1,2}$, are likely involved in the excessive and dysregulated inflammatory reactions elicited to ebolavirus infection, and thus likely also contribute to viral pathogenicity.

## SHED GP

Shed GP is a product of the proteolytic cleavage of cell surface $GP_{1,2}$ by TNF-α-converting enzyme (TACE) (Dolnik et al., 2004) (Figure 1). Shed GP is released from the cell surface in significant amounts in a soluble trimeric form (Dolnik et al., 2004). The proteolytic cleavage results in the removal of a short transmembrane anchor from the GP2 subunit of $GP_{1,2}$ (Figure 1). Due to the preservation of its antigenic properties, shed GP can be recognized by anti-$GP_{1,2}$ antibodies and exhibits a decoy function, sequestering anti-$GP_{1,2}$ antibodies that would otherwise bind to $GP_{1,2}$, and potentially induce the elimination of viruses and infected cells (Dolnik et al., 2004).

Recently, shed GP was shown to trigger the activation of non-infected dendritic cells and macrophages, leading to profuse expression of inflammatory cytokines, in a similar manner to $GP_{1,2}$, (Escudero-Perez et al., 2014). Interestingly, anti-TLR4 antibodies can efficiently block shed GP-mediated activation of immune cells; moreover, treatment of shed GP with either deglycosylases or mannose-binding lectin can inhibit the activity of shed GP (Escudero-Perez et al., 2014). These results suggest that cellular TLR4 signaling and shed GP surface glycans are likely involved in the inflammatory activation associated with ebolavirus infection. In addition, like $GP_{1,2}$, shed GP may also activate LSECtin signaling and trigger pro-inflammatory cytokine secretion by dendritic cells (Zhao et al., 2016).

Furthermore, treatment of HUVEC monolayers with shed GP increases their permeability, indicating a direct effect of shed GP in damage of the endothelial barrier (Zhao et al., 2016). Combined with the significant activities of some inflammatory cytokines, particularly TNF-α (Wahl-Jensen et al., 2005b), in impairment of endothelial barrier integrity, these data suggest that the disruption of endothelial barriers can be mediated directly by shed GP itself, by an unknown mechanism, or indirectly by shed GP-induced inflammatory cytokines (Escudero-Perez et al., 2014).

Additionally, aside from the RNA-editing strategy for regulation of GP expression (Volchkov et al., 2001), the release of shed GP may be another mechanism to modulate the abundance of cell surface $GP_{1,2}$, and hence virion $GP_{1,2}$ content and cytotoxicity, and this process likely has a role in orchestrating optimal ebolavirus infectivity and spread (Dolnik et al., 2015; Mohan et al., 2015).

## SOLUBLE GLYCOPROTEIN (sGP)

sGP, the primary product of the *GP* gene, is expressed from transcripts with no reading frame shift and is initially synthesized as pre-sGP (Volchkov et al., 1995;

Sanchez et al., 1996; Volchkova et al., 1999) (Figure 1). Pre-sGP then undergoes post-translational proteolytic cleavage and glycosylation, yielding mature sGP and a Δ-peptide (Volchkova et al., 1999) (Figure 1). sGP monomers can be dimerized in a parallel orientation to form a 110 kDa homodimer (Barrientos et al., 2004; Falzarano et al., 2006; Pallesen et al., 2016) (Figure 1). Both sGP and Δ-peptide can be secreted extracellularly, while Δ-peptide is retained in producer cells for a longer period than sGP (Volchkova et al., 1999).

Previous studies have suggested that sGP may exhibit versatile immunomodulatory functions (Kindzelskii et al., 2000; Ito et al., 2001; Sui and Marasco, 2002; Wahl-Jensen et al., 2005b; Mohan et al., 2012; de La Vega et al., 2015). Since sGP is the main product of the *GP* gene and shares a common N-terminus (295 aa residues) with full-length GP, it was hypothesized that, like shed GP, sGP acts as a decoy antigen by adsorbing the antibodies against $GP_{1,2}$, thus counteracting antibody-mediated clearance of viral infection (Wilson et al., 2000; Ito et al., 2001). Furthermore, a model of sGP-mediated "antigenic subversion" was proposed, which postulated that the much higher quantity of sGP may lead to sGP domination of host humoral responses, and divert the immune response away from $GP_{1,2}$ (Mohan et al., 2012).

sGP may also exhibit some anti-inflammatory activities, including inactivation of neutrophils and partial restoration of the TNF-α-induced decrease of the barrier function of endothelial cells (Kindzelskii et al., 2000; Sui and Marasco, 2002; Wahl-Jensen et al., 2005b), although the molecular mechanisms underlying these phenomena have not yet been determined.

In addition to being extracellularly secreted as a nonstructural protein, sGP appears to be able to assemble with GP2 as a substitute for GP1 and, intriguingly, the sGP-GP2 complex can also mediate infection in a vesicular stomatitis virus (VSV) pseudotype model, indicating a potential role for sGP as a structural protein (Iwasa et al., 2011). However, the biological significance of the replacement of GP1 by sGP in $GP_{1,2}$ has yet to be determined.

## Δ-PEPTIDE

Δ-peptide was first described by Volchkova et al. during the course of an investigation of the processing and maturation of sGP (Volchkova et al., 1999). sGP is N-glycosylated, while Δ-peptide is O-glycosylated (Volchkova et al., 1999) (Figure 1). The production of Δ-peptide is conserved across all ebolavirus species, suggesting an important role (or roles) for the peptide.

Radoshitzky et al. reported that Δ-peptides of several pathogenic ebolaviruses (EBOV, Sudan virus, and Taï Forest virus) can efficiently bind to filovirus-permissive cells and inhibit Marburg virus (MARV, another filovirus) and ebolavirus $GP_{1,2}$-mediated cell entry (Radoshitzky et al., 2011), even though MARV does not produce Δ-peptides. In contrast, the Δ-peptide of Reston virus exhibits less-efficient cell binding and no obvious inhibitory effect on cell entry of filoviruses, indicating a potential role for Δ-peptide in determining viral virulence (Radoshitzky et al., 2011). Suppression of ebolavirus entry by Δ-peptide, which is a post-infection product, may prevent virus superinfection and thereby benefit viral spread and systematic infection. Although the mechanism of Δ-peptide-mediated inhibition of filovirus infection remains unclear, the impairment of cell entry of both MARV and ebolaviruses by Δ-peptides suggests that these peptides probably interfere with a common pathway involved in filovirus entry (Radoshitzky et al., 2011).

Recently, Gallaher and Garry performed sequence analyses and computational modeling to identify a conserved amphipathic region in the Δ-peptides of filoviruses (particularly EBOV) with high similarity to the cytolytic peptide motif of rotavirus nonstructural protein 4 (NSP4) (Gallaher and Garry, 2015). The authors hypothesized that Δ-peptides may serve as membrane-damaging viroporins, thus contributing to ebolavirus pathogenesis (Gallaher and Garry, 2015). The membrane-association potential of Δ-peptides may explain the retention of Δ-peptides in cells after secretion of the bulk of sGP has occurred (Gallaher and Garry, 2015); nevertheless, whether Δ-peptides exhibit such biological functions requires experimental verification.

## SMALL SOLUBLE GLYCOPROTEIN (ssGP)

The existence of ssGP, which is encoded by the + 2-shift reading frame of the *GP* gene, had long been postulated before being experimentally verified by Mehedi *et al.* (Mehedi et al., 2011). The similar molecular masses of sGP and ssGP, the low abundance of ssGP (encoded by < 5% of *GP* transcripts) (Figure 1), and the lack of ssGP-specific antibodies make the detection of ssGP expression difficult by conventional methodology (Mehedi et al., 2011). To date, the function of ssGP is unknown. ssGP is essentially a truncated form of sGP and the two proteins share an identical N-terminal sequence (295 aa) (Figure 1). Moreover, mature ssGP is extensively N-glycosylated and forms homodimers, in the same way as sGP (Mehedi et al., 2011) (Figure 1). However, despite similarities in the biochemical properties and primary sequences of sGP and ssGP, ssGP does not demonstrate the anti-inflammatory activity exhibited by sGP that reverses TNF-α-induced loss of endothelial barrier function (Mehedi et al., 2011; Wahl-Jensen et al., 2005b).

## CONCLUSION

Underestimating highly virulent pathogens, such as EBOV, would be immensely costly to society. The 2014 Ebola outbreak not only resulted in devastating health effects and pronounced socio-economic impacts in West Africa, but also caused a global panic, particularly with the occurrence of imported Ebola cases in countries out-

Table 1. Summary of the known or potential functions of ebolavirus GPs

| GPs | Functions | References |
|---|---|---|
| Full-length GP ($GP_{1,2}$) | Mediates virus entry as the virion surface spike | Lee and Saphire., 2009 |
| | Promotes virus budding by antagonizing tetherin | Kaletsky et al., 2009 |
| | Sterically shields the epitopes and functions of cellular surface proteins via the MLD, causing rounding and detachment of cultured cells, endothelial cell damage, leakage of explanted blood vessels, and loss of cell physiological functions (such as antigen presentation by MHC-I) | Chan et al., 2000; Takada et al., 2000; Yang et al., 2000; Simmons et al., 2002; Reynard et al., 2009; Francica et al., 2010 |
| | Sterically shields the epitopes of the $GP_{1,2}$ core via the MLD, blocking recognition by neutralizing antibodies (?) | Reynard et al., 2009; Francica et al., 2010 |
| | Activates MΦ/DCs and triggers the secretion of inflammatory cytokines by the MLD (?), likely contributing to the excessive inflammation in EVD | Wahl-Jensen et al., 2005a; Ye et al., 2006; Martinez et al., 2007 |
| | Contains a putative ISD, mediating T cell dysfunction/apoptosis (?) | Volchkov et al., 1992; Yaddanapudi et al., 2006 |
| Shed GP | Functions as a decoy for anti-$GP_{1,2}$ antibodies, contributing to viral immune evasion | Dolnik et al., 2004 |
| | Activates MΦ/DCs leading to the secretion of inflammatory cytokines; increases the permeability of HUVEC monolayers | Escudero-Perez et al., 2014 |
| | Its release modulates the abundance of surface $GP_{1,2}$, likely orchestrating virus cytotoxicity, infectivity, and spread (?) | Dolnik et al., 2015 |
| | Contains a putative ISD, mediating T cell dysfunction/apoptosis (?) | Volchkov et al., 1992; Yaddanapudi et al., 2006 |
| sGP | Functions as a decoy of anti-$GP_{1,2}$ antibodies, or mediates "antigenic subversion", diverting the immune response away from $GP_{1,2}$ (?) | Wilson et al., 2000; Ito et al., 2001; Mohan et al., 2012 |
| | Inactivates neutrophils and reverses TNF-α-induced injury of endothelial barriers, playing anti-inflammatory roles (?) | Kindzelskii et al., 2000; Sui and Marasco., 2002; Wahl-Jensen et al., 2005b |
| | Assembles with GP2 as a substitute for GP1, perhaps as a structural protein (?) | Iwasa et al., 2011 |
| Δ-peptide | Binds to filovirus-permissive cells and inhibits filovirus $GP_{1,2}$-mediated cell entry | Radoshitzky et al., 2011 |
| | Contains an amphipathic region similar to the cytolytic peptide motif of rotavirus NSP4 and may serve as a membrane-damaging viroporin (?) | Gallaher and Garry., 2015 |
| ssGP | Unknown; unlike sGP, does not display the anti-inflammatory activity that reverses TNF-α-induced damage of endothelial barriers | Mehedi et al., 2011 |

Notes: GP, glycoprotein; GPs, glycoproteins; sGP, soluble glycoprotein; ssGP, small soluble glycoprotein; MLD, mucin-like domain; MHC-I, major histocompatibility complex class I; MΦ, macrophages; DCs, dendritic cells; EVD, Ebola virus disease; ISD, immunosuppressive domain; HUVEC, human umbilical vein endothelial cell; TNF-α, tumor necrosis factor-α; NSP4, nonstructural protein 4; "(?)" indicates putative functions that especially require additional verification.

side of Africa (U.S. Centers for Disease contrOl and Prevention, 2016b). Effective preventive and therapeutic methods are urgently needed to tackle this virus. Considering their essential roles in virus entry and multifaceted functions in viral pathogenesis (summarized in Table 1), GPs (especially $GP_{1,2}$) of ebolaviruses are important, and potentially preferred targets, for the prevention and treatment of ebolavirus infections, for example as targets for potential therapeutic antibodies (Qiu et al., 2014; Bornholdt et al., 2016; Corti et al., 2016; Furuyama et

Figure 2. Model for the roles of $GP_{1,2}$, shed GP, and sGP in ebolavirus pathogenesis. $GP_{1,2}$, shed GP, and sGP constitute the main GP products of ebolaviruses and are more fully characterized functionally than other GPs. Severe ebolavirus infections are typified by uncontrolled virus infection and spread, and systemic inflammation, to which $GP_{1,2}$, shed GP, and sGP likely contribute in various ways. In addition to the essential roles of $GP_{1,2}$ in the virus life cycle of facilitating virus entry and assembly, all three GPs are involved in virus immune escape, leading to uncontrolled disseminated virus replication and consequent increased GP production. $GP_{1,2}$ antagonizes tetherin to promote virus budding, mediates steric blocking of cell surface immune molecules (such as MHC-I) and perhaps the $GP_{1,2}$ core via the extensively O-glycosylated MLD, and may induce T cell dysfunction/apoptosis via the putative immunosuppressive domain (ISD) adjacent to the TACE cleavage site (indicated by a box). Shed GP retains the ISD and thus has the potential capacity to target T cells. Shed GP and sGP may both inhibit anti-$GP_{1,2}$ antibody-mediated clearance of viral infection by acting as decoy antigens. Moreover, sGP may also exert the same effect by inducing "antigenic subversion". On the other hand, $GP_{1,2}$ and shed GP both activate macrophages (MΦ) and dendritic cells (DCs) and induce inflammatory cytokine secretion by triggering TLR4 and LSECtin signaling pathways. In addition, $GP_{1,2}$ directly induces CPE, including cell rounding and detachment, and impairs cell physiological functions by blocking cell surface molecules with the MLD, leading to endothelial damage and vascular leakage and other tissue/organ injury. Shed GP can also directly cause increased endothelial permeability by an undefined mechanism. Both the direct toxicity of $GP_{1,2}$ and shed GP, and their inflammation-mediated pathogenic characteristics, may eventually contribute to the clinical manifestations of EVD, such as fever, hemorrhage, shock, and multi-organ dysfunction.

al., 2016; Howell et al., 2016; Misasi et al., 2016; Pallesen et al., 2016; Wec et al., 2016). Among the GP variants, sGP, $GP_{1,2}$, and shed GP are the main viral GP products and their functional roles are more fully characterized. We propose a model for the critical roles of $GP_{1,2}$, shed GP, and sGP in virus infection and pathogenesis, which is summarized in Figure 2. In addition to the direct roles of $GP_{1,2}$ in the virus life cycle, the three GP variants are all believed to contribute to ebolavirus immune evasion by multiple strategies, thus contributing to uncontrolled virus infection and spread (Figure 2). Moreover, $GP_{1,2}$ and shed GP may also promote pathological lesions and the development of clinical manifestations of EVD such as high fever, hemorrhage, shock, and organ dysfunction, by their direct effects (such as cytotoxicity) and the indirect effects induced by excessive inflammation (Figure 2).

Despite the important achievements of studies of ebolavirus GPs, many key facets of the functions of these proteins remain to be elucidated. For example, although many investigations have indicated that $GP_{1,2}$ can induce cytotoxicity and vascular damage, to date there is no *in vivo* data verifying these roles of $GP_{1,2}$. The physiological relevance of the observed functions of GPs *in vitro* needs to be further investigated *in vivo* using animal models, along with various *GP* gene-engineered recombinant ebolaviruses (Groseth et al., 2012). Functional and structural studies of the soluble GPs, especially ssGP and Δ-peptide, are relatively scarce, compared with those of $GP_{1,2}$. In addition to $GP_{1,2}$ and the soluble GPs discussed above, other products of the *GP* gene have also been identified (Sanchez et al., 1998; Volchkov et al., 1998b); these include the short transmembrane anchor remaining after the release of shed GP, secreted GP1 (separate from GP2), and lone GP2. Whether these proteins are merely by-products of the *GP* gene or have significant biological functions requires further investigation.

Since the majority of studies of ebolaviruses have focused on EBOV, the molecular biology and pathogenesis of ebolaviruses are best characterized for this species; however, we consider that the notable variation in the virulence of different ebolavirus species provides an opportunity to unravel viral pathogenesis, and that comparative studies of different species and diverse hosts would be of interest and are merited. Additionally, insights from ebolaviruses will be valuable in enabling better understanding of the biology and pathogenesis of other pathogens causing hemorrhagic fever, such as MARV and Crimean-Congo hemorrhagic fever virus (a bunyavirus), which also encode GPs with a heavily O-glycosylated MLD (Sanchez et al., 2006; Wertheim and Worobey, 2009). It is reasonable and interesting to hypothesize that these GPs and MLDs may play similar significant roles in the virulence and pathogenesis of these various pathogens.

## ACKNOWLEDGMENTS

This work was supported by the National Natural Science Foundation of China (No. 31125003 and No. 31321001) and the Basic Work Program of the Ministry of Science and Technology of China (2013FY113500).

## COMPLIANCE WITH ETHICS GUIDELINES

The authors declare that they have no conflicts of interest. This article does not contain any studies with human or animal subjects performed by any of the authors.

## OPEN ACCESS

This article is distributed under the terms of the Creative Commons Attribution 4.0 International License (http://creative commons.org/licenses/by/4.0/), which permits unrestricted use, distribution, and reproduction in any medium, provided you give appropriate credit to the original author(s) and the source, provide a link to the Creative Commons license, and indicate if changes were made.
